# The Interplay of Conjugation and Metal Coordination in Tuning the Electron Transfer Abilities of NTA-Graphene Based Interfaces

**DOI:** 10.3390/ijms23010543

**Published:** 2022-01-04

**Authors:** Magdalena Kaźmierczak, Bartosz Trzaskowski, Silvio Osella

**Affiliations:** Chemical and Biological Systems Simulation Lab, Centre of New Technologies, University of Warsaw, Banacha 2C, 02-097 Warsaw, Poland; m.kazmierczak@cent.uw.edu.pl (M.K.); b.trzaskowski@cent.uw.edu.pl (B.T.)

**Keywords:** DFT, graphene, chemisorption, self-assembled monolayer, work function, charge transfer

## Abstract

An artificial leaf is a concept that not only replicates the processes taking place during natural photosynthesis but also provides a source of clean, renewable energy. One important part of such a device are molecules that stabilize the connection between the bioactive side and the electrode, as well as tune the electron transfer between them. In particular, nitrilotriacetic acid (NTA) derivatives used to form a self-assembly monolayer chemisorbed on a graphene monolayer can be seen as a prototypical interface that can be tuned to optimize the electron transfer. In the following work, interfaces with modifications of the metal nature, backbone saturation, and surface coverage density are presented by means of theoretical calculations. Effects of the type of the metal and the surface coverage density on the electronic properties are found to be key to tuning the electron transfer, while only a minor influence of backbone saturation is present. For all of the studied interfaces, the charge transfer flow goes from graphene to the SAM. We suggest that, in light of the strength of electron transfer, Co^2+^ should be considered as the preferred metal center for efficient charge transfer.

## 1. Introduction

The inevitable depletion of fossil fuels is a fact, and becoming energy independent from them is an increasingly pressing problem. What really needs to be considered is a source of green energy capable of converting renewable or non-exhaustive resources in an efficient way. One of the approaches adapted in recent years is to harvest what nature perfected, namely, the photosynthetic process, in a laboratory setting. Although the processes governing natural photosynthesis are known, their reproduction on a laboratory scale is very complex, since it requires building up key components of the process without the lipid membrane present in the natural process, in a non-physiological environment [1]. Despite these difficulties, biodevices based on artificial photosynthesis have been produced with encouraging results for energy conversion and water splitting processes [2,3,4,5]. One of the main challenges of these interfaces is the formation of sufficiently strong connections between the electrode and the light harvesting protein which will ensure efficient charge transfer between the two parts of the device, without losing the protein activity. These connections can be created, for example, by incorporating small, organic molecules that serve as a linker between the peptide and the electrode surface [6,7]. In this approach, tailored, controllable linkers can be used to create a layer with adjustable properties that facilitate electron/hole transfer from one end of the device to the other. On the other hand, the electrode material must also possess suitable properties that will grant excellent electron transfer, and, without a doubt, graphene fulfills most of the requirements. Graphene is a flat and defect-free 2D material consisting of a single layer of carbon atoms arranged in a honeycomb structure, which as an electrode material has outstanding properties, including transparency, extraordinary mechanical strength, flexibility, exceptional electrical conductivity, and the ability to adsorb molecules on its surface [8,9,10,11,12,13]. 

There are, however, several obstacles that must be overcome before using this unique 2D material in any (bio)electronic device. In order to create a real working device, a band gap, even narrow, must be opened. For this purpose, and to create efficient charge transfer conditions between graphene and protein in the biodevice, it is crucial to design molecules that inhibit charge recombination while at the same time guaranteeing excellent direct electron transfer (DET). The introduction of a self-assembled monolayer (SAM) of small organic molecules on the graphene surface has proven to be a valuable strategy for creating interfaces with dynamic electron transfer and exceptional electronic properties for a variety of applications, such as biosensors [14,15,16,17,18,19,20,21,22], bioelectronics [17,18,23] or biofuel cells [14,23].

The general approaches to such modifications involve two main strategies. The first consists of the physical adsorption of molecules on the surface of graphene, which maintains its conjugated structure [15,16,17], as well as efficient charge mobility [23,24]. The drawback of this approach is the fact that non-bonding interactions (such as van der Waals, π–π, hydrophobic) are weaker than chemical bonds and may not ensure interface stability with the risk of losing the functionalization over time. The second strategy, namely chemisorption, leads to a durable connection thanks to the creation of a chemical bond between graphene and the molecule, ensuring the high stability of the system and preserving the electron transfer characteristic [25,26]. Yet, in this way, local disruptions of the graphene lattice (due to the formation of the chemical bond and the sp^3^ hybridization) might lead to a substantial reduction of the charge mobility. According to our previous studies, pyrene-nitrilotriacetic acid derivatives coordinated with metal dicatations, such as Ni^2+^ or Co^2+^ have proven to be a promising choice when creating a physisorbed SAM-graphene interface with a controllable direction of electron flow [27,28].

**Figure 1:**
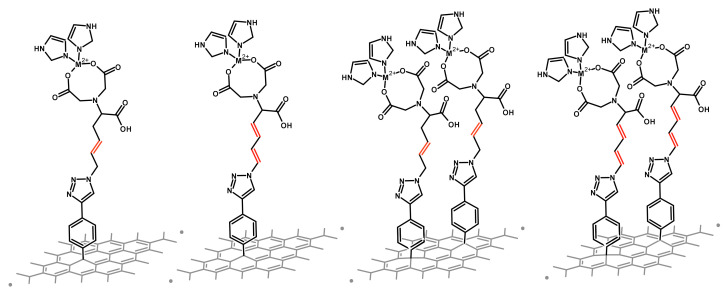
Schematic representation of the interfaces with two types of backbones studied in this work. Discussed double bonds are marked in red. The first two pictures are interfaces with 50% of coverage, the next two depict fully covered interfaces (100% coverage).

In the current work, we use quantum mechanical calculations to describe the electronic properties of chemisorbed SAM-graphene interfaces with modifications of the metal nature, backbone saturation, and surface coverage density (Figure 1). Modifications are introduced within the self-assembled monolayer molecules based on nitrilotriacetic acid moiety, which has been utilized before for experimentally obtained biosensors [29,30,31,32,33]. We discuss in detail changes in the work functions shifts (and its components) together with an in-depth charge transfer analysis upon the interface formation.

## 2. Results and Discussion

Throughout the study, we use the (M-XDB)_n_ notation to define the SLG-SAM interface, where M is Ni^2+^, Co^2+^, Cu^2+^ or PNTA (for interfaces without cation center), X is the number of double bonds (either one or two), and n is the number of SAM molecules in the supercell (explicitly marked when *n* = 2). It is worth noting that while two of the studied systems are formal radicals (Ni-1DB and Ni-2DB), the rest have a ground state triplet.

The present study is divided into two sections. First, we describe the effect of chain saturation on the work function shift by examining the properties of SAM molecules with one and two double bonds in the backbone when different metal ions are considered. In the second section, we investigate the effect of surface density on the WF and DET abilities of the interfaces by increasing the number of SAM molecules in one supercell.

### 2.1. Effect of Different Chain’s Saturation

#### 2.1.1. Interfaces without a Metal

The geometry of the PNTA-1DB and PNTA-2DB interfaces is strongly dependent on changes in the saturation of the backbone. While the first interface, with one double bond, is strongly bent by 50.6 degrees and forms an “L-shaped” structure, the presence of two double bonds causes a return to a linear structure that is only slightly tilted (7 degrees) with respect to the normal of the surface (see Supplementary Materials Figure S1). The adsorption energy is calculated by subtracting the energies of the isolated SAM molecule and the graphene layer from the total energy of the interface:

(1) $$E_{\mathrm{ads}}=E_{\mathrm{interface}}-E_{\mathrm{SAM}}-E_{\mathrm{SLG}}$$

Energies obtained for PNTA-1DB and PNTA-2DB are found to be −1.81 and −1.87 eV, respectively. Stable chemisorption of the SAM molecules to the graphene shifts the work function of graphene by +80 meV for PNTA-1DB and −320 meV for PNTA-2DB. In order to explain those results, the two components of work function shift, φ_SAM_ and φ_BD_, are analyzed. The presence of one double bond in the backbone leads to significant changes in the geometry of the system and the direction of the dipole moment, which eventually contributes to the positive value of φ_SAM_ of 120 meV. On the other hand, an additional double bond in the backbone causes the straightening of the structure, changes the direction of the dipole moment, and finally leads to a negative molecular contribution, equal to −260 meV. For both interfaces, the bond dipole contribution is rather low with a value range of −40/−60 meV. This rationalizes the values of Δφ, which are mainly driven by the dipole moment of the molecules. It is also worth noting that for the PNTA-1DB, work function shift contributions counteract, whereas for PNTA-2DB a cooperative effect is observed.

To rationalize the molecular contribution values, the dipole moments of isolated SAM molecules (calculated in a large cell to avoid intermolecular interactions) are taken into consideration and are found to be equal to −1.47 and 1.27 Debye, for molecules with one and two double bonds in the backbone, respectively. Dipole moments are strongly affected by the depolarization effect while forming the graphene interface, leading to a decrease to −0.29 and 1.12 Debye for PNTA-1DB and PNTA-2DB, respectively. The changes in magnitude and direction of the dipole moments for these two interfaces rationalize the differences in the molecular contribution, which, combined with the negligible bond dipole contribution, explain the changes in the work function shift.

The formation of a chemical bond between SAM and graphene creates a radical interface from which a semi-occupied crystal orbital (SOCO) is present. SOCO for both PNTA-1DB and PNTA-2DB is pinned to the Fermi level of the interface at −3.21 and −3.05 eV, respectively. Together with the lowest unoccupied crystal orbitals (LUCO), which energies are −1.86 and −1.73 eV, lead to a band gap opening of 1.35 and 1.32 eV, respectively. For both interfaces, the peak assigned to the highest occupied crystal orbital (HOCO) is delocalized over both graphene and SAM. On the other hand, while for PNTA-1DB the LUCO is localized on graphene, for PNTA-2DB a larger delocalization over both graphene and SAM has been found, most likely caused by the saturation along the whole molecular backbone (see Supplementary Materials Figures S2 and S3). For both interfaces, the SOCO is localized over the graphene-SAM bond.

For the discussed interfaces, partial charge transfer (CT) between the fragments is found. CT at the interface is described as the difference between the charge density of the whole system and the charge densities of its components (graphene and SAM). Such a subtraction will be referred to as an excess of electrons:

(2) $$\Delta \rho =\rho_{\mathrm{interface}}-\rho_{\mathrm{SAM}}-\rho_{\mathrm{SLG}}$$

For PNTA-1DB the depletion of electrons on the SAM equals 0.05 |e|, whereas for PNTA-2DB the excess of electrons on the SAM amounts to −0.06 |e|.

As a result, a different level of saturation of the backbone without the metal center shows the ability of PNTA molecules to modify the graphene work function in different ways. Introducing one double bond into the molecule greatly disturbs its geometry and results in a minor, positive work function shift of 80 meV leading to a charge transfer from the SAM to graphene. On the contrary, the presence of two double bonds negatively shifts the work function at a value of −320 meV, which translates into decreasing the electron injection barrier and easing the flow of electrons from graphene to the SAM.

#### 2.1.2. Interfaces with a Metal—One Double Bond

In this section, the effect of the backbone saturation on the properties of M-XDB interfaces is considered, while modifying the nature of the coordinating metal center (either Ni, Co, or Cu). The backbone of all three SAM-forming molecules with one double bond is bent with the inflection point located on the first carbon atom following the triazole ring (see Figure S4). This leads to a non-linear structure where the coordinated metal is tilted from the interface normal at about 35°. The adsorption energies equal −2.23 eV, −2.48 eV, and −2.45 eV for Ni-1DB, Co-1DB, and Cu-1DB, respectively, which indicates the formation of a C-C covalent bond between the graphene monolayer and the SAM.

**Figure 2:**
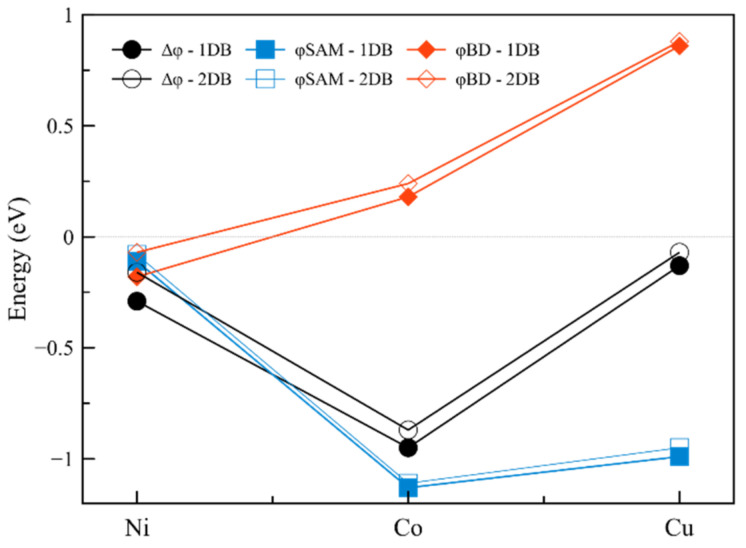
Work function shifts (black dots) and its two contributions: molecular, φ_SAM_ (blue squares) and bond dipole, φ_BD_ (red diamonds) of nickel, cobalt and copper interfaces with one (filled symbols) and two double bonds (empty symbols).

The work function changes from 4.38 eV for Ni-1DB, through 4.19 eV for Co-1DB and finally 4.17 eV for Cu-1DB, leading to a negative work function shift of −290, −950 and −130 meV for Ni-1DB, Co-1DB, and Cu-1DB, respectively (Figure 2, black line).

The strongest φ_SAM_ contributions are obtained for Co-1DB (φ_SAM_ = −1130 meV) and Cu-1DB (φ_SAM_ = −990 meV), which is in line with the high dipole moment calculated for SAM molecules in the absence of graphene (Table 1). Replacing the above metals with nickel results in a strong reduction of the dipole moment by more than 3 Debye and hence the molecular contribution to the work function shift. This is due to the different nature of the metal center; in fact, when Ni^2+^ is present, a radical is formed, thus leading to a weaker dipole moment. For both Co-1DB and Cu-1DB, the bond dipole (φ_BD_) contribution has a positive value, which causes the reduction of the work function shift, as it counteracts the backbone contribution. The strongest φ_BD_ contribution is found for Cu-1DB, with the value of φ_BD_ = 860 meV, almost counterbalancing the φ_SAM_ contribution, and therefore, strongly reducing the total work function shift. This effect is an indicator of a possible charge recombination mechanism at the interface when Cu is considered. A small positive value of φ_BD_ = 130 meV for Co-1DB causes a reduction in Δφ, however, not as strong as for the Cu-1DB interface, resulting in the highest work function shift. The opposite behavior is observed for Ni-1DB, for which both φ_SAM_ and φ_BD_ have negative values, most likely caused by the radical nature of the metal coordination center, thus both acting together to increase the magnitude of the work function shift.

As mentioned above, the dipole moment of the molecule is the main factor that influences the molecular backbone contribution to the work function shift, which is clearly visible when the interfacial dipole moments of the SAM (without graphene) are taken into consideration. The decrease in the value of the dipole moment when switching from cobalt (3.97 D) through copper (3.47 D) to nickel (0.38 D) is reflected in the decreasing contribution of the molecular backbone component (see Figure 2 and Table 1). The considerably low dipole moment of Ni1DB may be caused by the radical electron positioning itself on graphene (as indicated by the localization of the SOCO), thus reducing its value compared to Co-1DB and Cu-1DB. It is important to consider the phenomenon of depolarization as well, which leads to a reduction of 95%, 51% and 60% in dipole moment between the molecule alone and when packed into the SAM is observed for Ni-1DB, Co-1DB and Cu-1DB, respectively.

**Figure 3:**
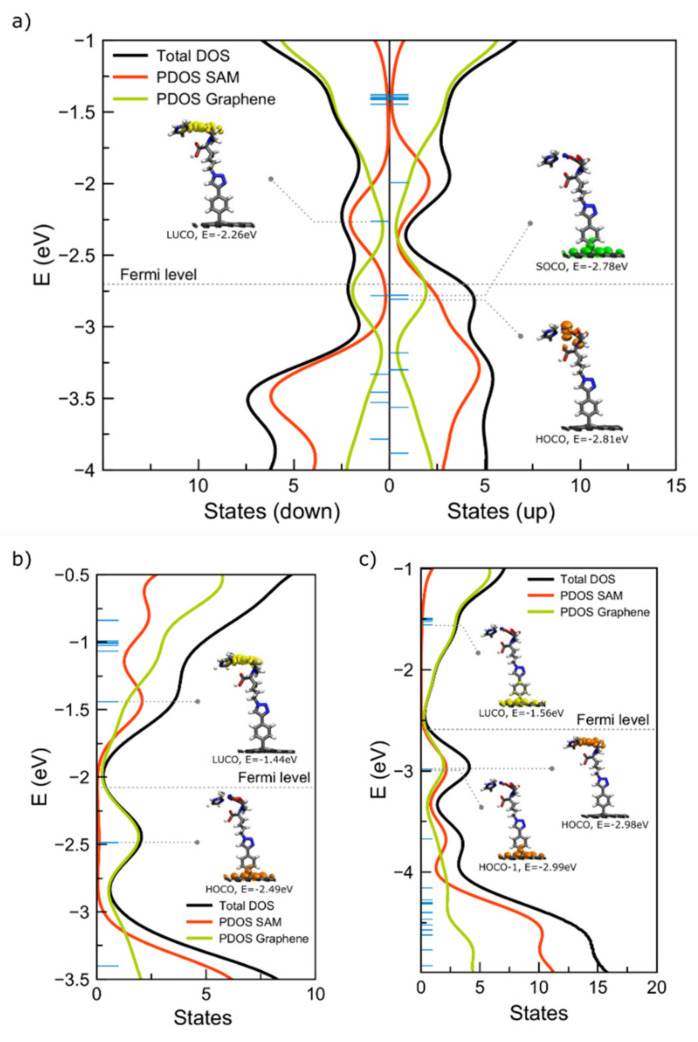
Total DOS projected over onto corresponding fragments for the Ni-1DB (**a**), Co-1DB (**b**) and Cu-1DB (**c**) interfaces. Eigenvalues are marked with horizontal blue lines and the Fermi level is indicated by a dotted grey line. HOCO (orange), SOCO (green) and LUCO (yellow) are marked and connected to corresponding eigenvalues.

In order to describe the energy levels of the interfaces, the density of states (DOS) analysis is performed. For Ni1DB, degeneration of both HOCO and SOCO is observed, with energies close to the Fermi level of −2.70 eV. Considerably close to SOCO is the LUCO energy (−2.26 eV), which leads to a rather small energy gap of 0.52 eV. The projected density of states (PDOS) indicates that both the HOCO and LUCO peaks have strong contributions from the SAM, while the SOCO is mostly located on graphene (Figure 3a). Indeed, for Ni-1DB visualization of MOs shows that both HOCO and LUCO are localized on the NTA-Ni moiety, while the SOCO is localized on graphene (Figure 3a).

A different picture emerges for the Co-1DB interface. Here, the HOCO at −2.49 eV is rather distant from the Fermi level (−2.08 eV). Consequently, LUCO energy of −1.44 eV, together with HOMO, opens a band gap of 1.05 eV. PDOS indicates a contribution of the HOCO peak arising from graphene and LUCO from SAM (Figure 3b). These results are fully confirmed by the different localizations of the molecular orbitals, for which the HOCO is located on graphene and the LUCO on the cobalt-NTA complex (Figure 3b).

The interpretation of the DOS and orbital shapes for Cu-1DB is more complex. Now, there are two degenerate HOCO orbitals, at −2.99eV. Together with LUCO at −1.56 eV, they open a band gap of 1.42 eV. Additionally, the HOCO orbitals of this system are located on opposite parts of the interface, which is shown in PDOS as two peaks, one on graphene and one on the metal complex (Figure 3c), while the LUCO is localized on graphene.

As mentioned above, all studied nickel interfaces are radical systems that led to the formation of SOCO (Figure 3b). For the Ni-1DB interface, the SOCO is localized on graphene, therefore, the electron transfer takes place from SLG to the SAM. The latter is confirmed by the charge density analysis, which indicates an electron excess of –0.02 |e| on the SAM. A slight enhancement of CT is observed for the Co-1DB interface, with a charge transfer of 0.03 |e| from SLG to SAM. A similar value of 0.02 |e| is obtained for Cu-1DB. For the latter, the excess of electrons is situated on the SAM part of the interface, suggesting electron transfer from the graphene slab onto the molecular backbone, and not vice versa, as the analysis of PDOS and MOs suggests. This unusual behavior is most likely caused by charge recombination induced by degeneration of the orbitals.

In summary, a strong dependency between the work function shift and the nature of the metal is observed. The formation of the Co-1DB interface results in the highest DET and work function shift. On the other hand, in light of the above results copper should be avoided as a coordination metal, because of the strong charge recombination observed.

#### 2.1.3. Interfaces with a Metal—Two Double Bonds

In the next step, we consider a SAM modification by introducing the second double bond to its backbone. Contrary to the systems discussed above, Ni-2DB, Co-2DB, and Cu-2DB do not possess an inflection point, but the entire SAM molecules in all three interfaces are tilted from the normal to the surface by approximately 17° (see Figure S5). The adsorption energies are equal to −2.17 eV, −2.46 eV, and −2.45 for Ni-2DB, Co-2DB, and Cu-2DB, respectively. These values are of the same magnitude as the ones obtained for one double bond system and ensure the creation of a stable SAM-graphene bond.

Despite the presence of a full conjugation along the SAM backbone, which should facilitate the electron transfer, the calculated work function exhibits no significant changes with the previously described systems, with values of 4.40 eV, 4.19 eV and 4.14 eV for Ni-2DB, Co-2DB and Cu-2DB, respectively. The corresponding work function shifts and contributions are shown in Figure 2. The highest work function shift is again found for the interface with cobalt, with a value of −870 meV, slightly lower compared to the corresponding one double-bond system. The same scenario is present also when the other metal cations are considered, with values of the work functions shift decreased by 45%, 8% and 46% for Ni-2DB, Co-2DB, and Cu-2DB, respectively. Considerably low decrease in Δφ may be linked to the low change in dipole moment (9%) when cobalt is present in the coordination center, contrary to the other two cations, for the which reduction of the dipole moment is considerably higher (47% for both nickel and copper).

Molecular and φ_BD_ contributions have similar trends compared to those obtained for the interfaces with one double bond. Namely, φ_SAM_ components (Figure 2, blue square contours) are decreased by 27%, 2%, and 4% for 2DB interfaces with nickel, cobalt, and copper, respectively, compared to the 1DB, which is most likely due to the alignment of the SAM to the interface normal and a slight decrease in the dipole moment (Table 2, φ_SAM_ column). Nevertheless, the high dipole moment obtained for SAM molecules for Co-2DB (3.88 D) and Cu-2DB (3.32 D) compared to Ni-2DB (0.29 D) is still the reason for the rather high molecular contribution for the first two interfaces and considerably low φ_SAM_ for the last one. Slightly different dependencies are found for φ_BD_. For Ni-2DB there is a decrease in the absolute value of the bond dipole contribution of 61% when the second double bond is introduced, while for the other two interfaces a general increase is found, of 33% for Co-2DB φ_BD_ and of 2% for Cu-2DB.

Upon analysis of DOS and molecular orbital localization, no significant changes are found when increasing the unsaturation from one to two double bonds, which is confirmed by the PDOS and the localization of the molecular orbitals for both Ni-2DB and Co-2DB (see Supplementary Materials Figures S6 and S7). An important change is the localization of the LUCO of the latter interface, which is now delocalized across the entire molecular backbone. The same holds true for the Cu-2DB interface, for which the unpaired electrons are located over two degenerated orbitals (HOCO and HOCO-1), one of which is localized over the graphene slab and the other on the molecular backbone of the SAM (see Supplementary Materials Figure S8).

Charge density analysis indicated an excess of electrons equal to 0.03 |e| on the SAM part of the interface for all three interfaces, with a charge flow direction from the graphene slab to the SAM. Double bond coupling may favor electron flow for interfaces with two double bonds compared to those with one bond, and result in an observed higher charge transfer value. The latter apparently slightly outweighs the effect of the metal.

The addition of a second conjugated bond to the backbone of the interface surprisingly leads only to minimal changes in the value of the work function shift and molecular and bond dipole contribution, in which the same trend is observed compared to that of the presence of one double bond. The greatest changes are observed when nickel is considered as a coordination center, for which Δφ, φ_SAM_ and φ_BD_ are reduced by 45%, 27% and 61%, respectively. Moreover, the DET direction is also not affected by the change in conjugation, pointing to a stronger effect due to the nature of the metal center rather than the number of double bonds present on the backbone. Therefore, from an electron transfer point of view, there is no significant difference between interfaces with one and two double bonds.

### 2.2. Effect of Surface Coverage Density

Finally, we evaluate the effect of surface covering on WF modification of the interfaces, by introducing a second SAM molecule into the same graphene supercell, to study the effects of depolarization (see Figure 1 and Figure S9 in Supplementary Materials). For all full-coverage interfaces, the work function value is higher than the corresponding half-coverage ones. For interfaces with Ni as the coordinating metal, the addition of a second molecule to the cell caused a significant increase in the absolute values of Δφ increasing it by 46% and 73% for (Ni-1DB)_2_ and (Ni-2DB)_2_. The same is observed going from (Cu-1DB)_2_ to (Cu-2DB)_2_, for which Δφ presents a fivefold increase for the first interface and sixfold for the second interface compared to the half coverage cases. Finally, both (Co-1DB)_2_ and (Co-2DB)_2_ have their work function shifts reduced compared to the half coverage situations, by 18% and 10%, and the final values are equal to −780 meV.

**Figure 4:**
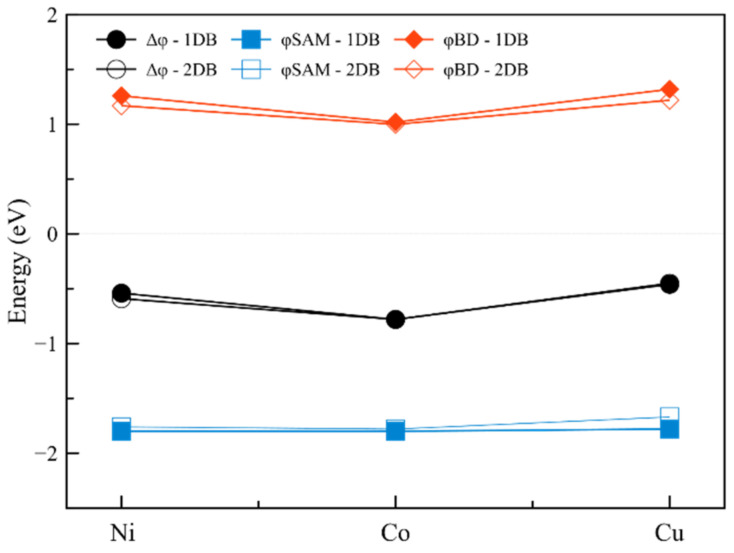
Total work function shift (black) and its two components: contribution of dipole moment of the molecule (blue) and contribution of bond dipole (red) for interfaces when two SAM-molecules are introduced to the graphene slab.

**Figure 5:**
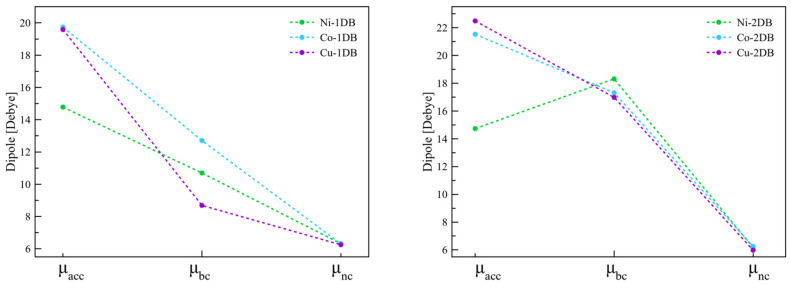
Depolarization shown for double interfaces. All three values were obtained for SAM-molecules in the absence of graphene layer with frozen structure. It is clearly visible that with growing intermolecular interaction the dipole moment decreases, causing significant shifts in φ_SAM_ values. For exact values see Table S1.

The contribution arising from the dipole moments of the backbones is approximately equal to −1.80 eV for all studied interfaces (Figure 4, blue lines), with the most outlaying value of −1.67 eV for (Cu-2DB)_2_. However, the highest increase in φ_SAM_, of 16 and 22 times, is observed for (Ni-1DB)_2_ and (Ni-2DB)_2_ respectively. This phenomenon can be explained when changes in dipole moments due to depolarization effects are taken into account. In order to clarify them, three parameters are considered: µ_acc_, µ_bc_ and µ_nc_ (µ_accumulated_, µ_big-cell_, µ_normal-cell_). µ_acc_ is obtained by summing the values of dipole moments calculated for isolated SAM molecules in the gas phase; µ_bc_ is calculated when two SAM molecules are placed together, inside an enlarged unit cell (i.e., dimer in the gas phase). Finally, µ_nc_ is obtained for the SAM molecules, when the size of the unit cell is retained. The reason why µ_acc_ has the highest values is the lack of intramolecular interactions within the molecules (Figure 5, µ_acc_). A strong depolarization caused by dimer interactions can be observed, hence the value of the µ_bc_ is lower than the corresponding value of µ_acc_ (Figure 5, µ_bc_). The highest reduction of 56% in dipole moments is observed for (Cu-1DB)_2_ (all depolarization values are reported in Table S2). Depolarization is even stronger when all intramolecular interactions are present. Considering the decreasing tendency in the values of dipole moments, it is clear that in the case of introducing the second molecule into the unit cell, depolarization plays the greatest role, eliminating all influences of the metal ion or the degree of saturation. Since for all systems the final dipole moment, µ_nc_, is practically identical, the molecular component will also have similar values. However, one cannot rule out the possibility that the rather small dimensions of the cell may contribute to the alignment of the dipole values, which might differ in a larger cell.

The leveling effect of full coverage is also reflected on the φ_BD,_ which now has nearly the same positive values. This behavior has the greatest impact for nickel-containing interfaces, since for (Ni-1DB)_2_ and (Ni-2DB)_2_ this contribution has a positive value, countering the negative value of φ_SAM_. Such behavior is completely different from that of the half-covered systems, where both contributions are negative. For (Co-1DB)_2_, (Co-2DB)_2_, (Cu-1DB)_2_ and (Cu-2DB)_2_ no change in φ_BD_ sign is observed, although there is an increase in its absolute value.

**Figure 6:**
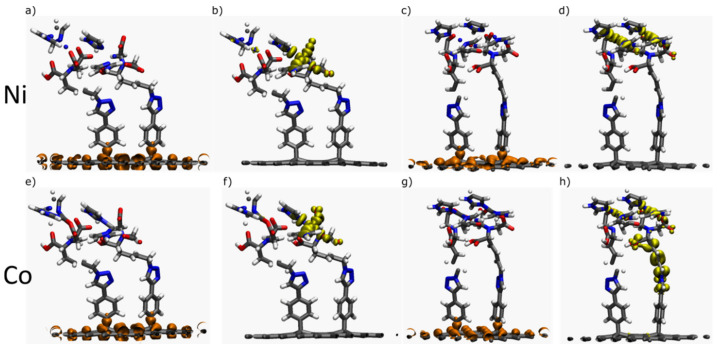
Shapes of HOCO (orange) and LUCO (yellow) molecular orbitals for (Ni-1DB)_2_ (**a**,**b**), (Ni-2DB)_2_ (**c**,**d**), (Co-1DB)_2_ (**e**,**f**) and (Co-2DB)_2_ (**g**,**h**). For DOS projected over onto corresponding fragments see Supplementary Materials (Figures S10–S15).

The addition of a second SAM molecule in the cell results in an increase of the energy of most of the orbitals (see Table S2 and Figures S10–S13 in Supplementary Materials). Increasing the coverage widens the band gap by 33%, 30%, and 50% for (Ni-1DB)_2_, (Co-1DB)_2_ and (Co-2DB)_2_ compared to half-covered interfaces but narrows it for (Ni-2DB)_2_ by 8%. For cobalt and nickel containing systems, PDOS shows similar orbital localizations; namely, for the HOCO peak, the main contribution arises from graphene, and for the LUCO from SAM. In this way, they resemble the pattern observed earlier for half-covered systems, which is also confirmed by MOs localization (Figure 6).

The band gap is drastically narrowed for both (Cu-1DB)_2_ and (Cu-2DB)_2_, by more than 75%, compared to Cu-1DB and Cu-2DB. Degeneration, previously observed for Cu-1DB and Cu-2DB, occurs also when the second SAM molecule is chemisorbed onto the graphene slab and leads to two degenerate HOCO states, one on the graphene and one on the SAM molecule (as confirmed by both PDOS and MOs localization, see Figures S14 and S15).

The charge density analysis for (Ni-1DB)_2_, (Co-1DB)_2_ and (Cu-1DB)_2_ shows an excess of electrons equal to 0.01 |e| for all three interfaces. This observation implies that increasing the coverage in the system reduces the DET to a significant extent throughout the interface with one double bond in the backbone. A different scenario arises when two double bonds are present in the backbone; for (Ni-2DB)_2_, (Co-2DB)_2_ and (Cu-2DB)_2_ the excess of electrons increases, and for the first two interfaces reaches 0.05 |e|. As is the case for half-covered systems, the charge transfer for fully covered nickel and cobalt interfaces is directed toward SAM. Although charge density analysis indicates an excess of electrons on the SAM part of the interface (which is in line with the position of LUCO), the presence of one of HOCOs on the same fragment of the system may lead to inhibited charge transfer for both (Cu-1DB)_2_ and (Cu-2DB)_2_ interfaces.

The discussed dependencies show the smoothing and equalizing effect of depolarization on the values of the work function shift and its components. For (Ni-1DB)_2_, (Ni-2DB)_2_, (Cu-1DB)_2_ and (Cu-2DB)_2_ a rise in Δφ is observed when the coverage is doubled. On the other hand, for (Co-1DB)_2_ and (Co-2DB)_2_ a decrease in work function shift is observed. Because of the depolarization phenomenon, the φ_SAM_ contribution is approximately equal for them all. The greatest impact in φ_BD_ is observed for (Ni-1DB)_2_ and (Ni-2DB)_2_ for which the introduction of the second SAM molecule into the cell not only increases the magnitude of the φ_BD_ value but also changes its sign. All the above leads to the conclusion that the effect of coverage far outweighs the effect of the metal and unsaturation in the creation of electronic properties of the interface.

## 3. Materials and Methods

The modeled system for our computational study is prepared considering 1-pentyl-4-phenyl-1H-1,2,3-triazole-nitrilotriacetic acid (PNTA), which is chemically bonded to a graphene surface and in which the saturation of the backbone is systematically altered. The nitrilotriacetic acid (NTA) moiety allows the coordination of an M^2+^ ion (where M–Ni, Co, Cu) and two imidazole molecules complete the first coordination shell (see Figure 1). In order to examine changes in the electronic properties when supramolecular effects are present, systems consisting of either one or two identical SAM molecules chemisorbed on the graphene supercell are studied. The supercell dimensions are set to allow for accommodation of two SAM-forming molecules, with cell parameters set at a = 10.70 Å, b = 6.179 Å, c = 50 Å, α = 120°, β = 90°. Vector *c* is set to 30–50 Å to create a vacuum area above the interface, and hence eliminate any interactions between the repeating units. To minimize the computational costs due to the large size of the system, geometry optimization is conducted by means of the tight-binding density functional theory (TB-DFT) method [34,35], using the DFTB+ software [34]. In this method, the superposition of the wave functions for isolated atoms is calculated to create an approximate set of wave functions for the considered system. This approach was previously successfully used for similar systems and was the starting point for calculations using more sophisticated methods with satisfactory accuracy [36,37,38]. The self-consistent distribution of the Mulliken charges (SSC) is implemented within the TB-DFT to improve the precision of the optimization and to initially describe the electrostatic properties of the system.

The optimized interfaces are subsequently analyzed with the Quantum Espresso 6.5 suite of programs [39]. Ultrasoft pseudopotentials [40] together with the PBEsol [41] functional and individually tuned cut-offs for wavefunctions and charge density for each metal (see Table S1 for details) are used. Dipole corrections are applied to maintain a constant, zero external electric field and minimize the electrostatic potential fluctuations, which could be disturbed by the formation of a graphene-SAM interface.

In order to characterize the electronic properties, such as electron transfer, the projected density of states (PDOS), molecular orbitals, work function, and averaged electrostatic potential along the normal to the surface are calculated. The work function is obtained as:

Δφ = φ_0_ – FL (3)

Where φ_0_ is the work function defined at the metal side of the interface and FL is the Fermi level of the interface. Moreover, the shift in the work function (Δφ) is calculated and consists of two factors: the dipole moment of the backbone (molecular contribution, φ_SAM_) and the electronic reorganization resulting from SLG—SAM bond formation (bond dipole contribution, φ_BD_):

Δφ = φ_SAM_ + φ_BD_ (4)

The first contribution is measured through direct calculation of the electrostatic potential profile of the SAM in the absence of graphene, whereas coordinates are kept constant. The φ_BD_ interaction is obtained by subtracting the molecular contribution from the total work function shift.

According to the Helmholtz model, the molecular contribution can be expressed by the formula below:

(5) $$\varphi_{\mathrm{SAM}}=\frac{e\cdot \mu_{\mathrm{SAM}}}{\varepsilon_{0}\cdot S}$$

Where μ_SAM_ is the dipole moment of SAM along the z-axis, *S* is the surface per molecule and $\varepsilon_{0}$ is vacuum permittivity [42,43]. Thus, the dipole directed towards the SAM molecule and metal complex causes a negative shift in the work function. If the dipole moment is directed towards graphene, this effect would be reversed causing the shift in work function to take positive values.

## 4. Conclusions

This work presents a theoretical study on SLG-NTA interfaces coordinated by metal cations, such as Ni^2+^, Co^2+^ or Cu^2+^ and their properties in the light of three effects: (i) type of the metal, (ii) backbone saturation, and (iii) surface coverage density. Increasing the level of saturation (without metal center) strongly affects the electronic properties of the interfaces, since the introduction of the double bonds to the system greatly influences the geometry of the system, as well as the charge transfer magnitude and direction. This is due to a reverse in the direction of the dipole moment of the SAM, going from one to two double bonds, which alters the WF shift direction, and thus the electrons flow.

A strong dependency between the value of work function shift and the type of metal in the coordination center is confirmed. Both cobalt and nickel ensure charge transfer, with a rather stronger flux of electrons for the first of these metals. Contrary to them, the presence of copper induced strong charge recombination, which in turn inhibits the CT. On the other hand, a saturation of the backbone, when the metal-NTA complex is present, slightly reduces the work function shift and its molecular contribution for all interfaces but does not have a more significant influence on the charge transfer.

Having considered the effects of the metal type and the saturation of the backbone, it is important to note the limitations imposed by the effect of the surface coverage density. Combining two SAM molecules in one cell leads to substantial depolarization and, hence, to decrease in the dipole moment values and limiting the WF shift. In such a way the effect of surface coverage outweighs the two effects previously discussed. On the other hand, increasing the number of molecules in the cell may promote DET when two double bonds are present in the backbone.

Given the advantages and disadvantages of M-XDB complexes outlined in the article, it is predictable that cobalt is the most reasonable choice for the coordination center since it provides both high Δφ and a considerable charge transfer. In summary, the current work is a comprehensive characterization of the electronic properties of compounds that paves the way for potential applications in biodevices and, as such, is the next step in the implementation of more complex interfaces.

## Figures and Tables

**Table 1:** Dipole moment, work function shift, molecular and bond dipole contributions of Δφ and charge transfer value for M-1DB interfaces.

	Interface	Dipole Moment of SAM [Debye]	Dipole Moment of the Interface [Debye]	Δφ [meV]	φ_SAM_ [meV]	φ_BD_ [meV]
1	Ni-1DB	0.38	1.01	−290	−110	−180
2	Co-1DB	3.97	3.34	−950	−1130	180
3	Cu-1DB	3.47	0.45	−130	−990	860

**Table 2:** Dipole moment, work function shift, molecular and bond dipole contributions of Δφ and charge transfer value for M-2DB interfaces.

	Interface	Dipole Moment of the Interface [Debye]	Dipole Moment of SAM [Debye]	Δφ [meV]	φ_SAM_ [meV]	φ_BD_ [meV]
1	Ni-2DB	0.54	0.29	−160	−80	−70
2	Co-2DB	3.04	3.88	−870	−1110	240
3	Cu-2DB	0.24	3.32	−70	−950	880

## Data Availability

Not applicable.

## Supplementary Materials

The following supporting information can be downloaded at: https://www.mdpi.com/article/10.3390/ijms23010543/s1.
