# Supplementary material for: The Interplay of Conjugation and Metal Coordination in Tuning the Electron Transfer Abilities of NTA-Graphene Based Interfaces
Source: Int J Mol Sci. 2022 Jan 4;23(1):543. doi: 10.3390/ijms23010543 (PMC8745182; doi:10.3390/ijms23010543)
Supplement: Supplementary file 1 [file ijms-23-00543-s001.zip › ijms-1532556-supplementary.pdf]

Supporting Information for

The interplay of conjugation and metal coordination in tuning the electron transfer abilities of NTA-graphene based interfaces

M. Kaźmierczak, B. Trzaskowski, S. Osella*

Chemical and Biological Systems Simulation Lab, Centre of New Technologies, University of Warsaw, Banacha 2C, 02-097 Warsaw, Poland.

Table S1. Cut-off values for single point calculations

	Ni^{2+}	Co^{2+}	Cu^{2+}	No metal
Ecutwfc [Ry]	90	65	50	50
Ecutrho [Ry]	480	455	380	200

Table S2. Parameters of the studied systems

	Ni				Co				Cu			
	1DB		2DB		1DB		2DB		1DB		2DB	
	MS	DS	MS	DS	MS	DS	MS	DS	MS	DS	MS	DS
System dipole (Debye)	1.01	1.89	0.54	2.08	3.34	2.75	3.04	2.75	0.45	1.62	0.24	1.58
SAM dipole (Debye)	0.38	6.32	0.29	6.17	3.97	6.31	3.88	6.25	3.47	6.25	3.32	5.86
Depolarization [%]	95	41	80	66	51	50	52	64	60	28	58	65
Depolarization 2 (if applicable) [%]	-	28	-	-24	-	36	-	20	-	56	-	24
Fermi level [eV]	-2.70	-2.12	-2.80	-2.14	-2.08	-1.76	-2.13	-1.81	-2.59	-2.19	-2.61	-2.31
Band gap [meV]	520	690	840	770	1050	1360	980	1460	1420	300	1350	350
E_{ads} [eV]	-2.23	-4.61	-2.17	-4.68	-2.48	-5.32	-2.46	-5.42	-2.45	-4.65	-2.45	-4.69
WF [eV]	4.38	4.48	4.40	4.50	4.19	4.63	4.19	4.74	4.17	4.47	4.14	4.51
WF shift [meV]	-290	-540	-160	-590	-950	-780	-870	-780	-130	-460	-70	-450
DV [eV]	-110	-1800	-80	-1760	-1130	-1800	-1110	-1780	-990	-1780	-950	-1670
BD [eV]	-180	1260	-70	1170	180	1020	240	1000	860	1320	880	1220
Charge transfer [|e|]	0.02	0.01	0.03	0.05	0.03	0.01	0.03	0.05	0.02	0.01	0.03	0.04
HOMO energy [eV]	-2.81	-2.27	-3.06	-2.30	-2.49	-2.07	-2.54	-2.16	-2.99	-2.34	-3.03	-2.45
SOMO energy or energy of second HOMO [eV]	-2.78	-	-2.88	-	-	-	-	-	-2.98	-2.34	-2.98	-2.45
LUMO energy [eV]	-2.26	-1.58	-2.04	-1.53	-1.44	-0.71	-1.56	-0.70	-1.56	-2.04	-1.63	-2.10
CT direction	SLG -> SAM								-			

1. Tilt angles for PNTA-1DB and PNTA-2DB

The tilt angle was calculated between the plane perpendicular to SLG and the metal centre.

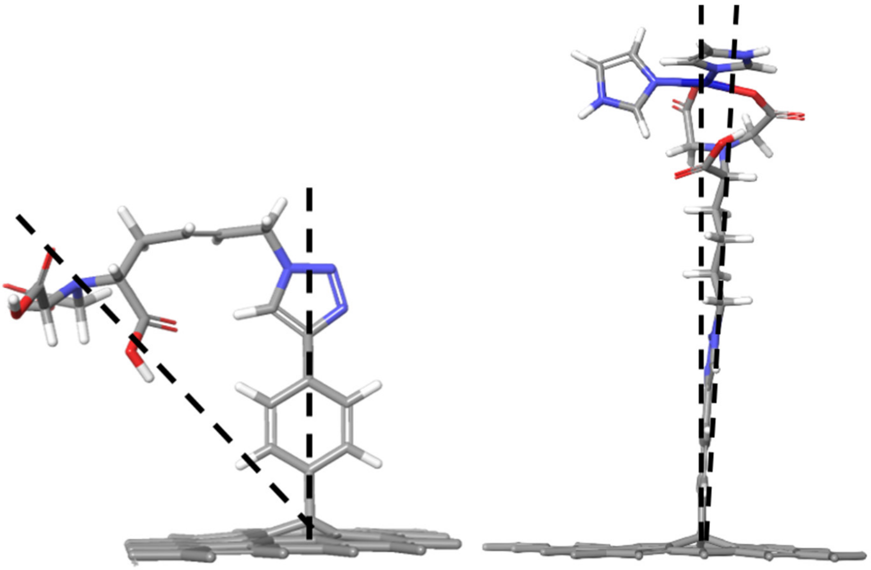

Figure S1. Schematic representation of the tilt angle calculation for PNTA-1DB and PNTA-2DB interfaces, respectively.

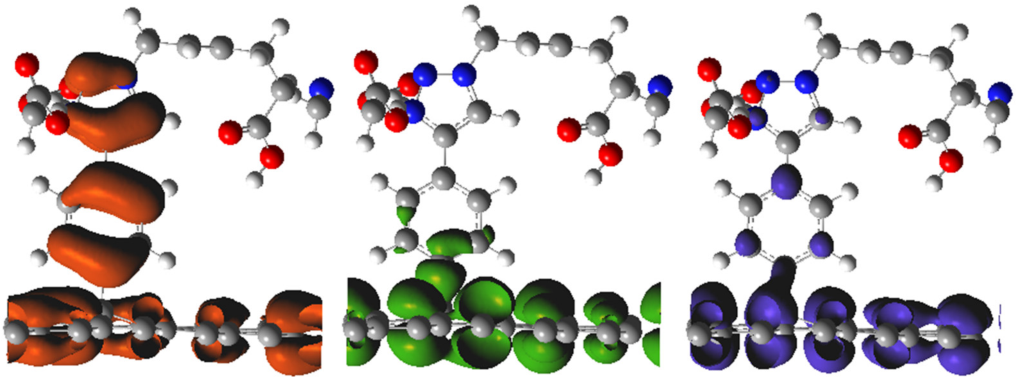

Figure S2. Frontier orbital analysis for the PNTA-1DB interface. Red colour refers to the HOCO, green to SOCO and blue to LUCO, respectively.

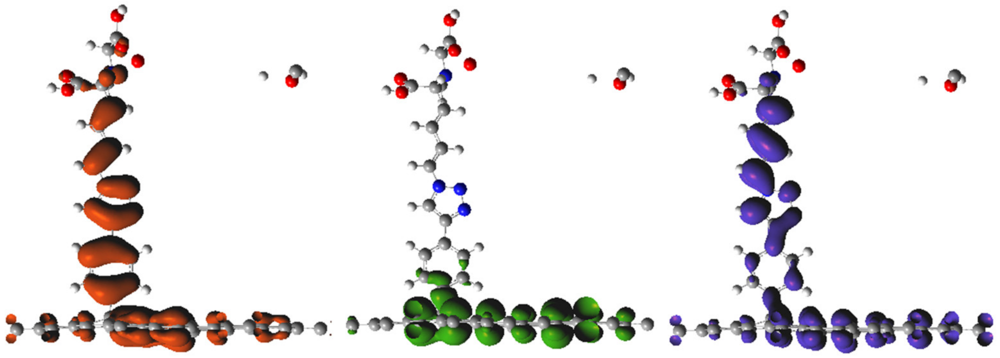

Figure S3. Frontier orbital analysis for the PNTA-2DB interface. Red colour refers to the HOCO, green to SOCO and blue to LUCO, respectively.

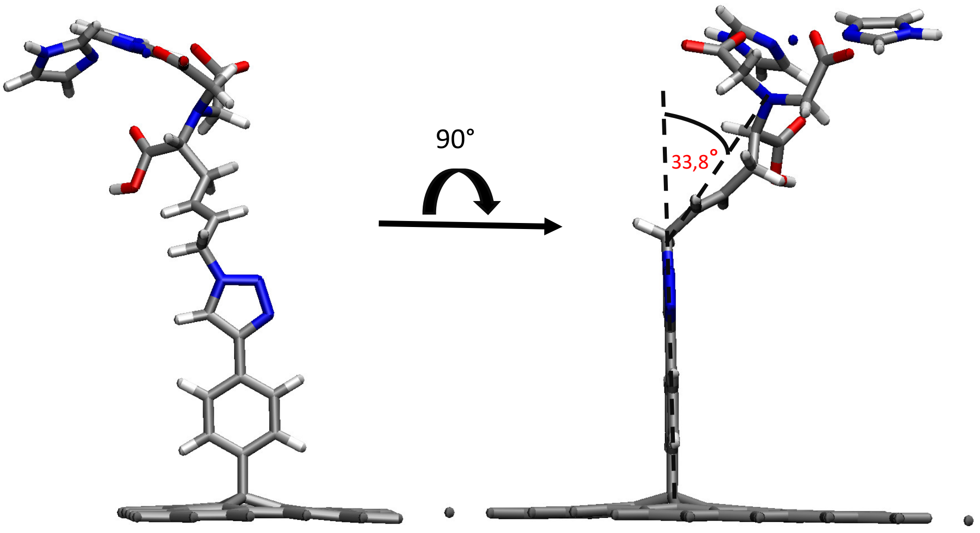

Figure S4. Schematic representation of the tilt angle for the X-1DB interfaces.

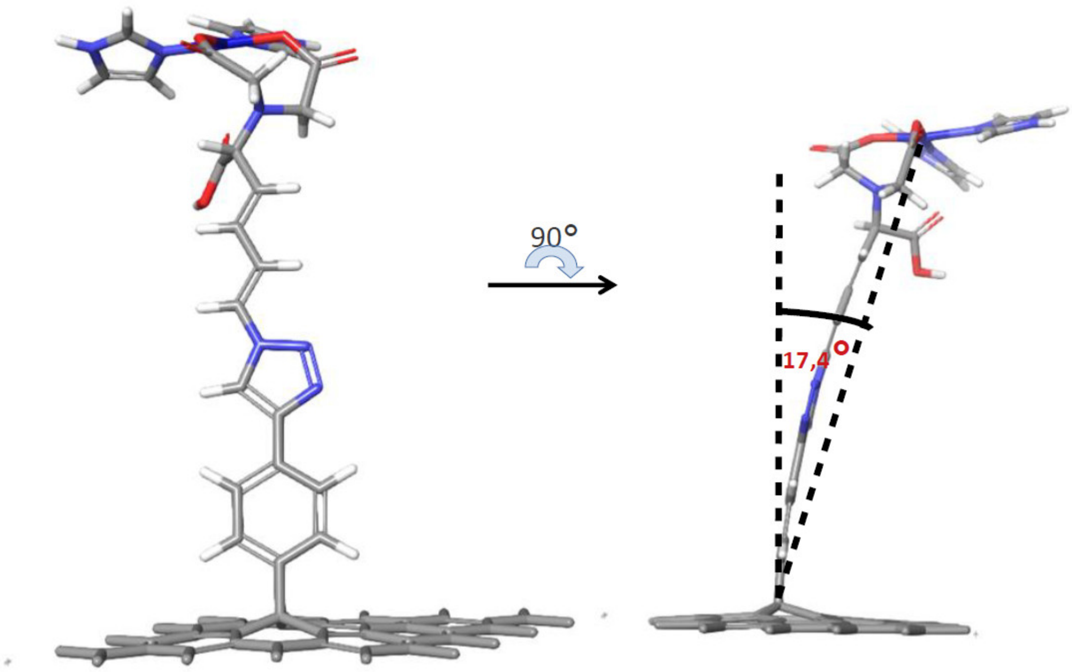

Figure S5. Schematic representation of the tilt angle for the X-2DB interfaces.

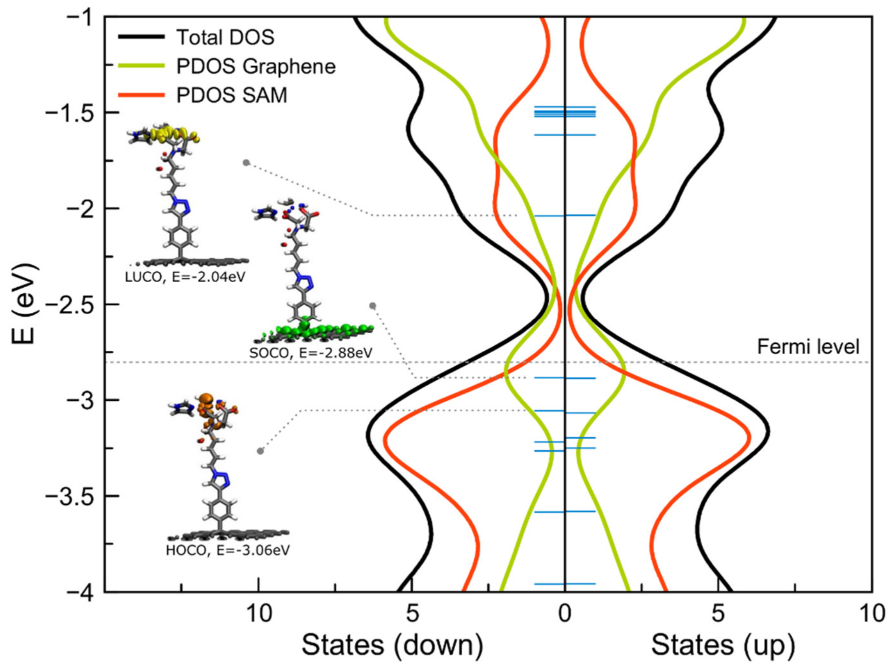

Figure S6. Total DOS projected over onto corresponding fragments for the Ni-2DB interface. Eigenvalues are marked with horizontal blue lines and the Fermi level is indicated by a dotted gray line. HOCO (orange), SOCO (green) and LUCO (yellow) are marked and connected to corresponding eigenvalues.

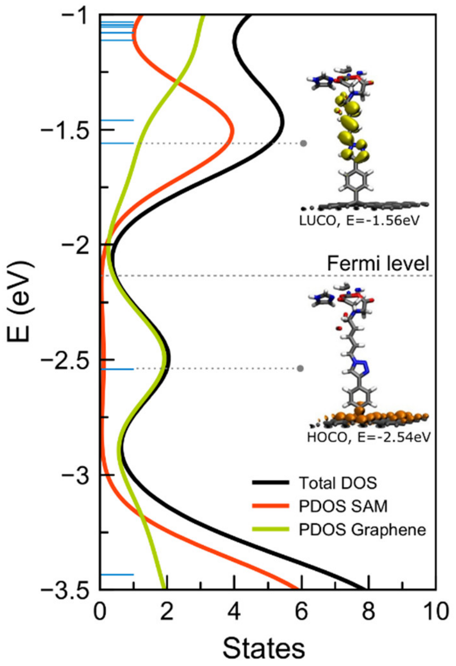

Figure S7. Total DOS projected over onto corresponding fragments for the Co-2DB interface. Eigenvalues are marked with vertical blue lines and the Fermi level is indicated by a dotted black line. HOCO (orange) and LUCO (yellow) are marked and connected to corresponding eigenvalues.

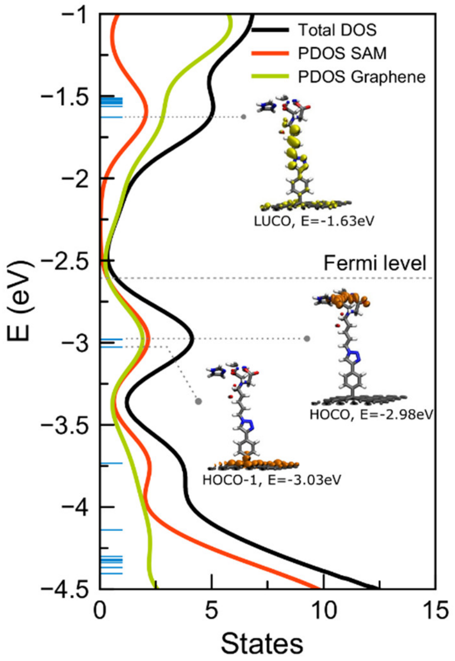

Figure S8. Total DOS projected over onto corresponding fragments for the Cu-2DB interface. Eigenvalues are marked with horizontal blue lines and the Fermi level is indicated by a dotted gray line. HOCO (orange) and LUCO (yellow) are marked and connected to corresponding eigenvalues.

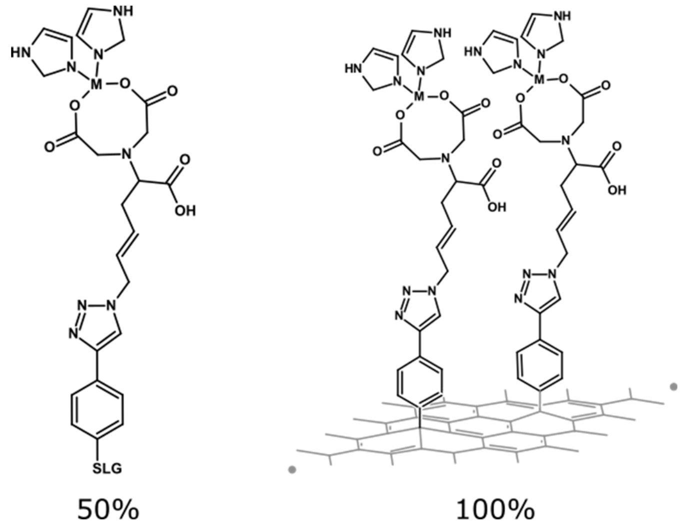

Figure S9. Schematic representation of half (left) and fully (right) coverage interfaces.

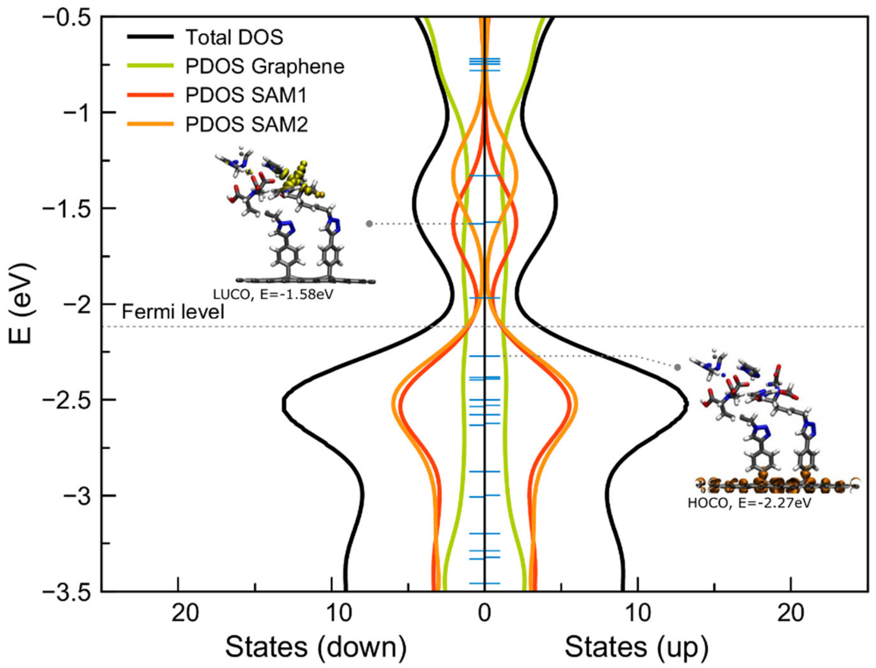

Figure S10. Total DOS projected over onto corresponding fragments for the (Ni-1DB)$_2$ interface. Eigenvalues are marked with horizontal blue lines and the Fermi level is indicated by a dotted gray line. HOCO (orange) and LUCO (yellow) are marked and connected to corresponding eigenvalues.

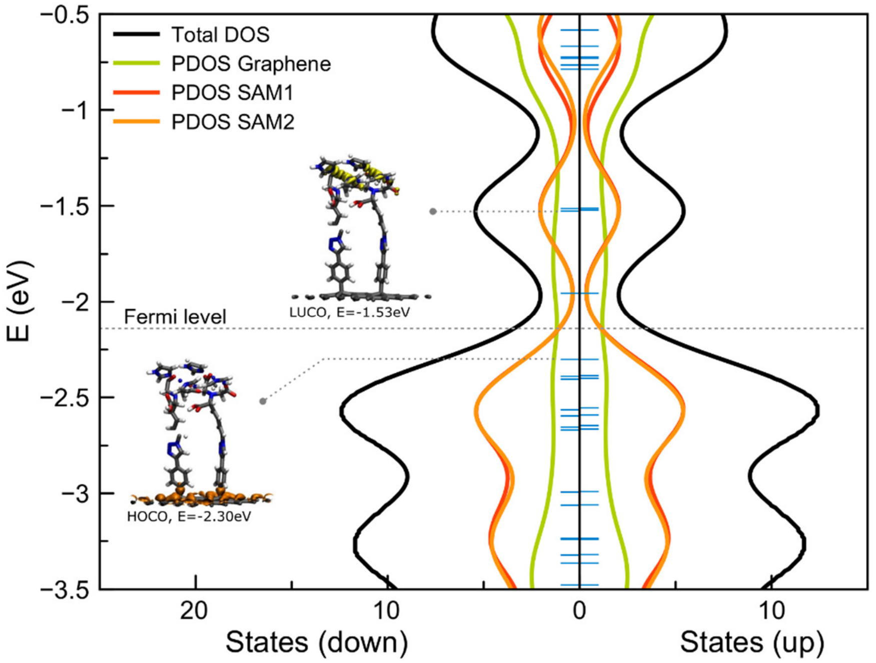

Figure S11. Total DOS projected over onto corresponding fragments for the (Ni-2DB)$_2$ interface. Eigenvalues are marked with horizontal blue lines and the Fermi level is indicated by a dotted gray line. HOCO (orange) and LUCO (yellow) are marked and connected to corresponding eigenvalues.

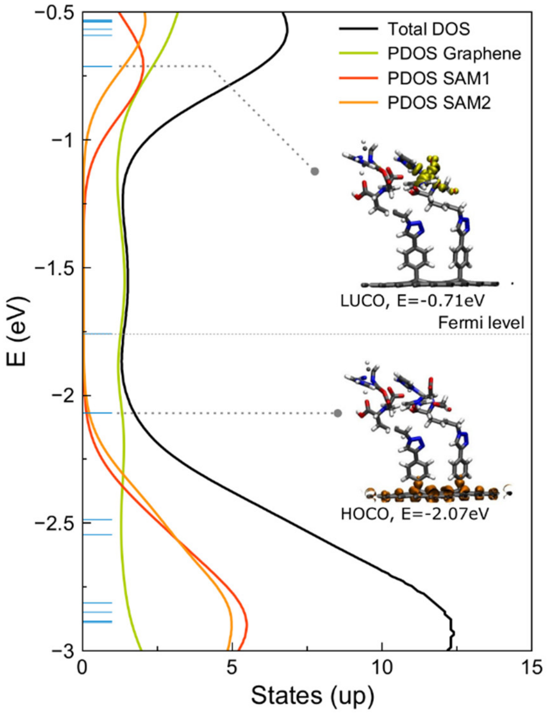

Figure S12. Total DOS projected over onto corresponding fragments for the $(Co\text{-}1DB)_2$ interface. Eigenvalues are marked with vertical blue lines and the Fermi level is indicated by a dotted black line. HOCO (orange) and LUCO (yellow) are marked and connected to corresponding eigenvalues.

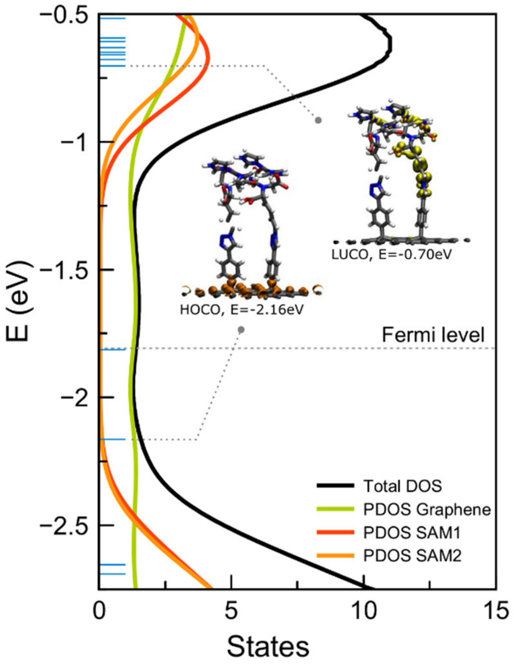

Figure S13. Total DOS projected over onto corresponding fragments for the $(Co\text{-}2DB)_2$ interface. Eigenvalues are marked with horizontal blue lines and the Fermi level is indicated by a dotted black line. HOCO (orange) and LUCO (yellow) are marked and connected to corresponding eigenvalues.

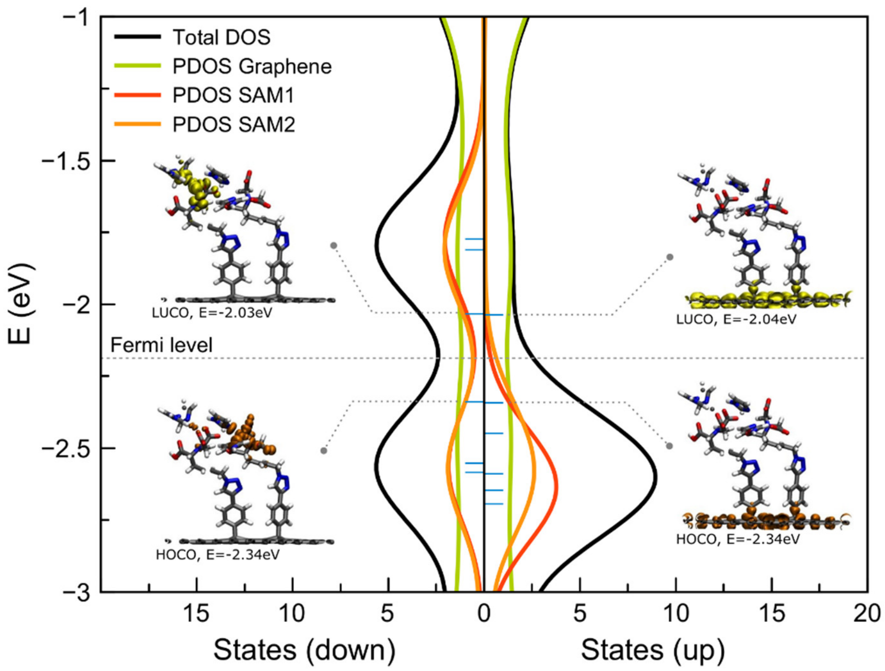

Figure S14. Total DOS projected over onto corresponding fragments for the (Cu-1DB)$_2$ interface. Eigenvalues are marked with horizontal blue lines and the Fermi level is indicated by a dotted gray line. HOCO (orange) and LUCO (yellow) are marked and connected to corresponding eigenvalues.

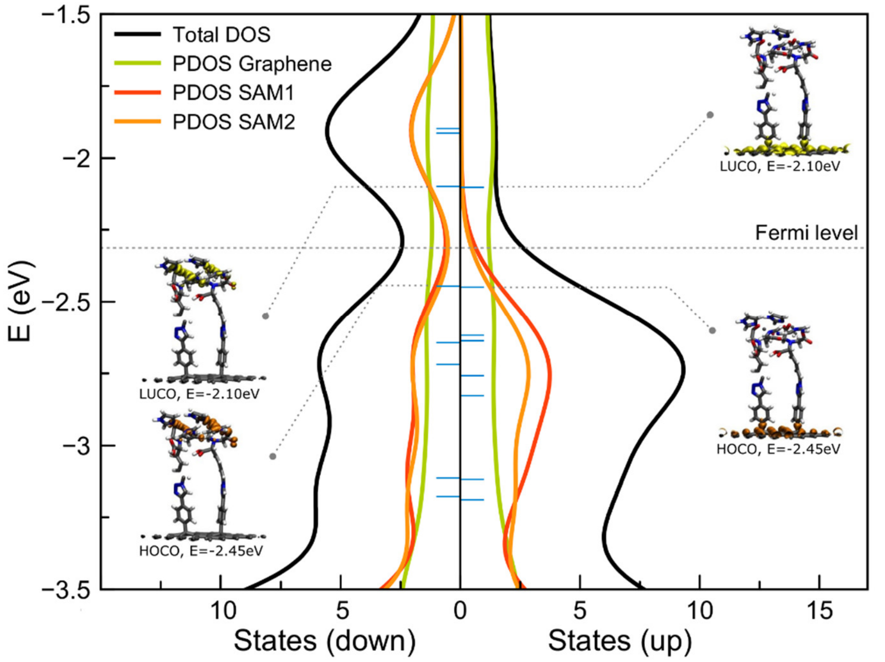

Figure S15. Total DOS projected over onto corresponding fragments for the (Cu-2DB)$_2$ interface. Eigenvalues are marked with horizontal blue lines and the Fermi level is indicated by a dotted gray line. HOCO (orange) and LUCO (yellow) are marked and connected to corresponding eigenvalues.
